# Structure and Properties of Polylactic Acid Biocomposite Films Reinforced with Cellulose Nanofibrils

**DOI:** 10.3390/molecules25143306

**Published:** 2020-07-21

**Authors:** Qianqian Wang, Chencheng Ji, Jianzhong Sun, Qianqian Zhu, Jun Liu

**Affiliations:** 1 Biofuels Institute, School of the Environment and Safety Engineering, Jiangsu University, Zhenjiang 212013, China; cecilji@amecnsh.com (C.J.); happyzqq@ujs.edu.cn (Q.Z.); junliu115142@ujs.edu.cn (J.L.); 2 State Key Laboratory of Pulp and Paper Engineering, South China University of Technology, Guangzhou 510640, China; 3 Analysis and Testing Center, Jiangsu University, Zhenjiang 212013, China

**Keywords:** cellulose nanofibrils, polylactic acid biocomposite, solution casting, melt compression, mechanical property, thermal property

## Abstract

Polylactic acid (PLA) is one of the most promising biodegradable and recyclable thermoplastic biopolymer derived from renewable feedstock. Nanocellulose reinforced PLA biocomposites have received increasing attention in academic and industrial communities. In the present study, cellulose nanofibrils (CNFs) was liberated by combined enzymatic pretreatment and high-pressure homogenization, and then subsequently incorporated into the PLA matrix to synthesize PLA/CNF biocomposite films via solution casting and melt compression. The prepared PLA/CNF biocomposite films were characterized in terms of transparency (UV-Vis spectroscopy), chemical structure (attenuated total reflectance-Fourier transform infrared, ATR-FTIR; X-ray powder diffraction, XRD), thermal (thermogravimetric analyzer, TGA; differential scanning calorimetry, DSC), and tensile properties. With 1.0–5.0 wt % additions of CNF to the PLA matrix, noticeable improvements in thermal and physical properties were observed for the resulting PLA/CNF biocomposites. The 2.5 wt % addition of CNF increased the tensile strength by 8.8%. The T_onset_ (initial degradation temperature) and T_max_ (maximum degradation temperature) after adding 5.0 wt % CNF was increased by 20 °C, and 10 °C, respectively in the nitrogen atmosphere. These improvements were attributed to the good dispersibility and improved interfacial interaction of CNF in the PLA matrix.

## 1. Introduction

Nanocellulose, which can be used as nanoscale reinforcing fillers, are known for their high aspect ratio, low density, large surface area, excellent mechanical performance, and low environmental impact [1,2]. Generally, nanocellulose can be classified into three different categories, namely, cellulose nanocrystals (CNCs), cellulose nanofibrils (CNFs), and bacterial cellulose (BC) based on their properties and isolation methodologies [3,4]. High-quality nanocellulose now can be produced not only in the laboratory but also in pilot plants [5,6]. The industrial productions of nanocellulose are also carried out in CelluForce (CNC, 300 ton/year), American Process (CNF and CNC, each 130 ton/year), Nippon Paper (CNF, 560 ton/year), and University of Maine (CNF, 260 ton/year) [6]. The advancement in nanocellulose production has laid the foundation for the large-scale application of nanocellulose as a reinforcing filler in thermoplastic. PLA was increasingly studied as packaging materials. However, the insufficient thermal, mechanical, and barrier properties of PLA limited its performance in various applications. One approach to solving these drawbacks is using nanocellulose as reinforcement fillers in the PLA matrix. Nanocellulose reinforced PLA biocomposites likely offer a bright future for PLA in the field of packaging [7].

PLA biocomposites reinforced with nanocellulose including CNC, CNF, and BC have been fabricated and extensibility investigated [8,9,10]. However, due to the polarity difference, good dispersibility of nanocellulose in the PLA matrix is challenging. Thus modifications of CNC or PLA are essential for the fabrication of PLA/nanocellulose biocomposites [11,12,13,14]. Unlike the rigid CNC whisker, CNF and BC exhibit long and flexible chains, which mediate possible entanglement with the PLA matrix. Many studies used the pristine CNFs and BC as reinforcement fillers without modification in PLA-based composites [12,15]. Melt-extrusion is one of the key processing methods for large scale production. Nanocellulose reinforced PLA biocomposites by the melt-processing techniques have been widely reported [16,17]. The agglomeration and thermal degradation of nanocellulose fillers in the melt-processing techniques are two urgent challenges to be solved. Solution casting was used in the laboratory to achieve good dispersibility for nanocellulose in the PLA matrix [18]. It was reported that the addition of a small percentage of microcrystalline cellulose undermined both the mechanical strength and barrier properties of PLA biocomposite due to the low level of dispersibility and interfacial interaction between microcrystalline cellulose and PLA matrix [19]. In most cases, the incorporation of a small amount of nanocellulose increased mechanical strength and thermal stability as compared to that of neat PLA matrix [20,21]. It was reported that the optimal loadings of nanocellulose in the PLA composites were only 0.5–2 wt % [12]. The synergistic nanocellulose and nanoclay reinforced PLA biocomposite films maintained high transparency, and exhibited improved thermomechanical properties and crystallization kinetics [22].

As discussed above, in some cases, the physical properties of PLA composites were significantly enhanced by nanocellulose incorporation, whereas, in other cases, no improvement and even a decrease in the physical properties of PLA composites were reported. This inconsistency was probably due to the preparation or modification methods of nanocellulose, and the level of dispersibility and interfacial adhesion. In our previous study, CNF was successfully isolated by combined enzymatic treatment and high-pressure homogenization. The performance of CNF isolated in our group has not been examined as reinforcement filler in the PLA matrix. Thus the effects of CNF isolated by combined enzymatic treatment and high-pressure homogenization on the optical, chemical, thermal, and mechanical strength of PLA/CNF biocomposites were examined in detail by UV-vis spectrophotometer, ATR-FTIR, XRD, TGA, differential scanning calorimetry (DSC), and tensile tester. Good dispersibility and nucleation effect of CNF isolated by combined enzymatic pretreatment and high-pressure homogenization may enhance the properties of PLA biocomposites.

## 2. Results and Discussion

### 2.1. Properties of Isolated CNF

**Figure 1:**
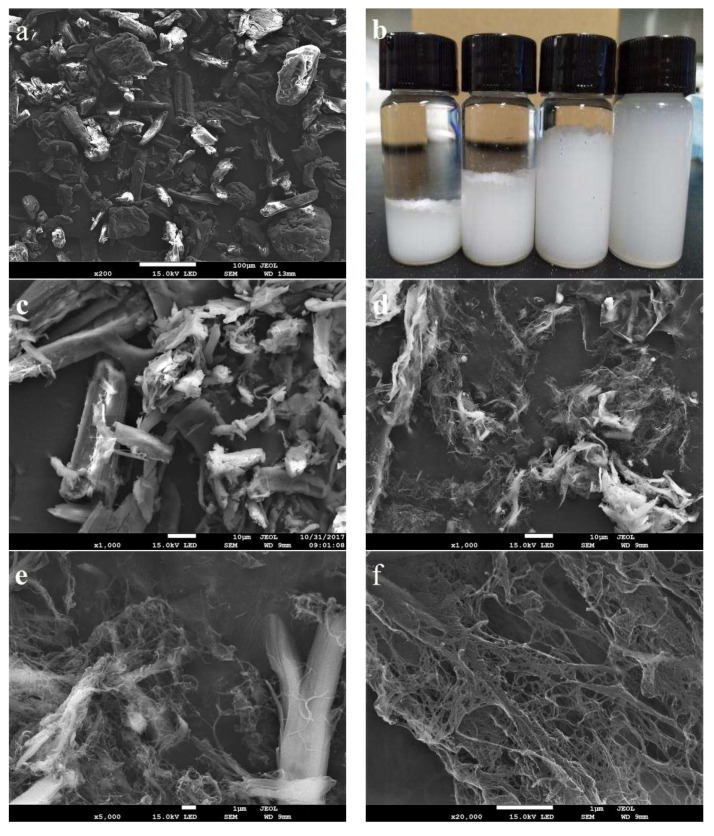
SEM images of MCC (**a**) and a freeze dried cellulose nanofibril (CNF) (**c**–**f**), and their visual appearance (**b**) after 30 days of storage: from left to right (**c**) enzymatic pretreated MCC; (**d**) CNF-2-pass, (**e**) CNF-5-pass, and (**f**) CNF-10-pass.

SEM images of the starting microcrystalline cellulose (MCC) and cellulase pretreated MCC are displayed in Figure 1a, c. Cellic^®^ CTec2, a complex cellulase cocktail with exoglucanase, endoglucanase, and lytic polysaccharide monooxygenases (LPMOs), provided extensive hydrolysis of less crystalline segments of MCC. Consequently, MCC was disrupted to fragments and fines as shown in Figure 1c. It was also reported that LPMOs ingredient in the cellulase cocktails might introduce some oxidation group on the CNF surface [23]. These oxidation groups may alter the interfacial properties between CNF and PLA matrix, which contribute to the good dispersibility and compatibility of the CNF filler in the PLA matrix. Enzymatic fibrillation further facilitated the breakdown of the MCC to CNF that have a significantly smaller length and width. SEM images in Figure 1d–f exhibit the morphological changes in the progressive homogenization process for 2, 5, and 10 passes. Enzymatic pretreated MCC was internally and externally fibrillated into fines or nanofibrils by mechanical shearing and compression during high-pressure homogenization. Some fibers were highly fibrillated into nanofibrils, whereas others were slightly fibrillated into a submicron size as shown in Figure 1d,e. It seems that further homogenization resulted in less large agglomerates and more nanofibrils fragmentation (Figure 1f). Figure 1b showed that the aqueous suspension of enzymatic pretreated MCC, CNF-2-pass, CNF-5-pass, and CNF-10-pass at the concentration of 2.0 wt % after 30-day storage, which further proved the degree of fibrillation. The dispersion stability of CNFs in water can also reflect the CNF size distributions. CNF-10-pass displayed excellent dispersion stability after long storage in water, which indicated more uniform and finer size distribution. Only CNF-10-pass was used in the subsequent study due to its excellent stability and more uniform size distribution.

**Figure 2:**
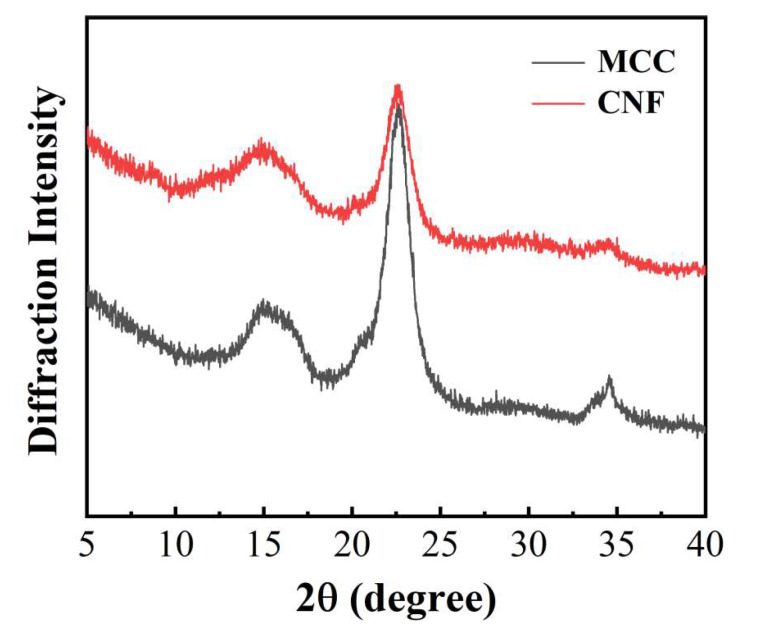
XRD patterns of MCC and CNF.

X-ray powder diffraction was adopted to analyze the crystal structure of MCC and CNF as shown in Figure 2. Both MCC and CNF exhibited typical cellulose I structure, which showed (1–10), (200), and (004) planes at 2*θ* = 15.1°, 22.6°, and 34.3°, respectively [24]. It was indicated that enzymatic pretreatment and homogenization did not alter the cellulose I structure of MCC and CNF. The (200) and (004) planes at 22.6° and 34° of CNF were dramatically weakened after enzymatic pretreatment and high-pressure homogenization. Although enzymatic pretreatment can increase the crystallinity index by removing less crystalline region in MCC, high-pressure homogenization largely destroyed the high crystalline region of MCC and increased the specific surface area of CNF, resulting in more amorphous regions. The crystallinity of MCC was 75.9%, while the crystallinity of CNF was 67.7% as determined by Segal’s method [25].

### 2.2. Optical Transmittance of CNF Filled PLA Biocomposite Films

**Figure 3:**
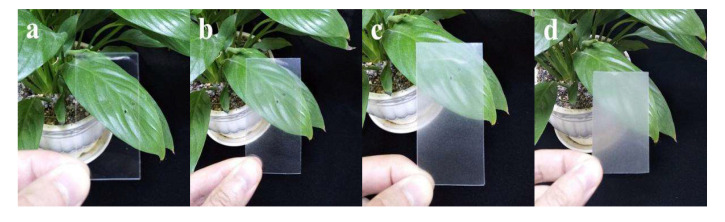
Visual images of PLA/CNF biocomposite films: (**a**) neat PLA; (**b**) PLA/CNF-1.0 wt %; (**c**) PLA/CNF-2.5 wt %; and (**d**) PLA/PLA/CNF-5.0 wt %.

**Figure 4:**
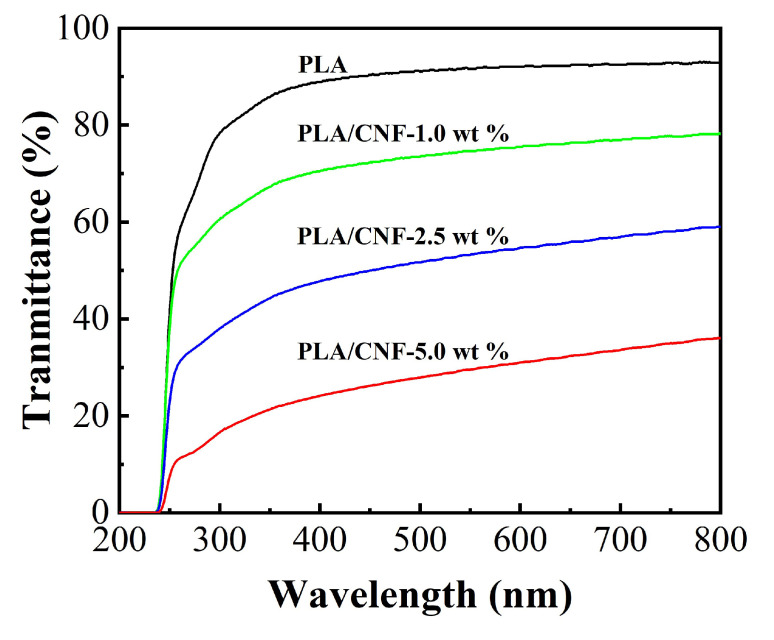
Optical transmittance of PLA/CNF biocomposite films with different CNF loading: neat PLA (black); PLA/CNF-1.0 wt % (green); PLA/CNF-2.5 wt % (blue); and PLA/CNF-5.0 wt % (red).

After the two-step process of solution casting and melt compression, smooth PLA/CNF composite films were obtained as shown in Figure 3. Pristine PLA film showed high transparency. PLA/CNF-1.0 wt % biocomposite films exhibited slightly lower transparency than that of neat PLA film. The transparency of PLA/CNF-2.5 wt % and PLA/CNF-5.0 wt % biocomposite films was much lower, which can be qualitatively reflected by checking the green plants behind. White spots in PLA/CNF biocomposites were visible in the study by Jonoobi et al. [26]. In our study, no white spots are detected even at the highest CNF loading (5.0 wt %), indicating better dispersibility in our PLA/CNF biocomposites. As shown in Figure 4, UV-Vis spectroscopy characterization of the biocomposite films was also performed and the results were consistent with the optical observation. PLA film exhibited high transparency in the wavelength range from 300 to 800 nm. The addition of CNF gradually reduced the transmittance of biocomposite films in both visible region and UV region.

### 2.3. FTIR and XRD Analysis of CNF Filled PLA Biocomposite Films

**Figure 5:**
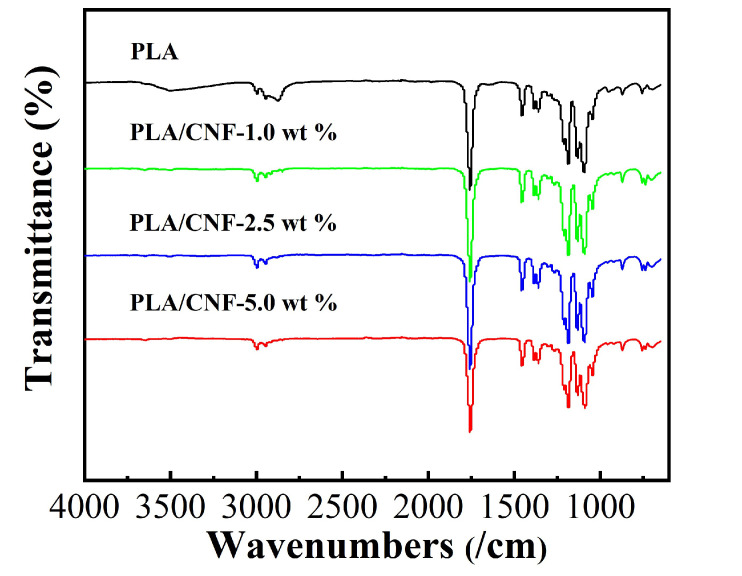
ATR-FTIR analysis of PLA/CNF biocomposite films.

The ATR-FTIR spectra for pure PLA and PLA/CNF biocomposite films with different CNF contents are shown in Figure 5. ATR-FTIR spectrum showed a strong absorption band at 1757 cm^−1^, which was attributed to the symmetrical stretching characteristic peak of carbonyl (-C=O) of the ester bond in PLA. The band at 1180 was ascribed to the C-O stretch in the CH-O- ester group. The band at 1080 cm^−1^ was -C-O- stretching vibration in the -O-C=O group. The bending vibration of -C-H- in CH_3_ groups and the main chain of PLA were detected at 1450 cm^−1^ and 1380 cm^−1^, respectively. These characteristics bands were also located in the literature [27,28]. PLA/CNF biocomposite films displayed all characteristic bands of PLA. No new bands were observed in PLA/CNF biocomposites than that of the characteristics bands for PLA. This may suggest there were mainly physical interactions between PLA and CNF. With the increase of CNF contents, ATR-FTIR spectra of PLA/CNF biocomposite films were indistinguishable from that of the pure PLA film.

**Figure 6:**
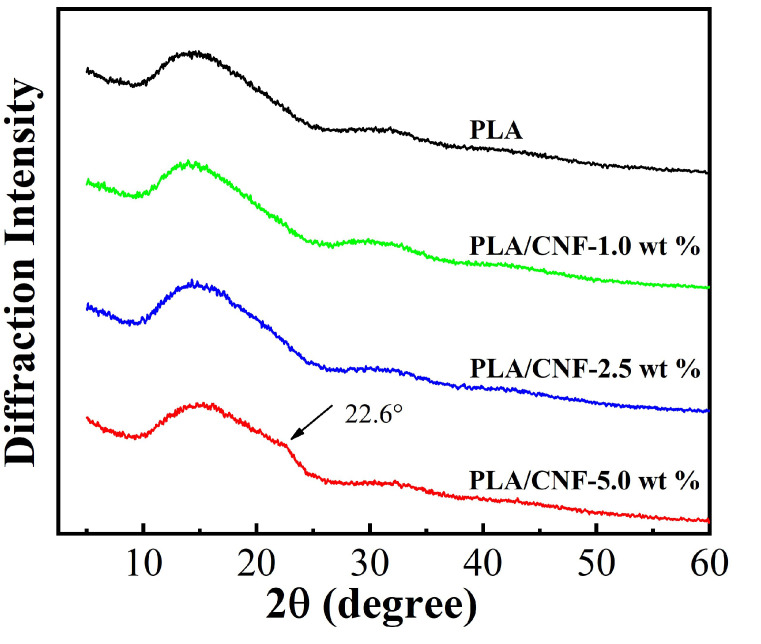
XRD patterns of PLA/CNF biocomposite films.

Neat PLA and PLA/CNF biocomposite films were characterized by XRD. The diffraction patterns were collected in the 2*θ* range of 4–40°, as shown in Figure 6. A broad characteristic peak was observed at 15° for the neat PLA film, indicating the semi-crystallization during processing [29]. The XRD spectra of the pure PLA and PLA/CNF-1.0 wt % acid are very similar, indicating that the semi-crystalline structure of PLA was not changed by CNF incorporation [30]. The CNF diffraction peaks were masked by the PLA diffraction peak. Similarly, no obvious difference was detected between pure PLA and PLA/CNF-2.5 wt % in the XRD patterns. A small shoulder in the diffraction pattern of PLA/CNF-5.0 wt % at about 22.6° was detected, suggesting the overall crystallinity increase in the biocomposite film due to the strong (200) peak of CNF as shown in Figure 2.

### 2.4. TGA Analysis of CNF Filled PLA Biocomposite Films

**Figure 7:**
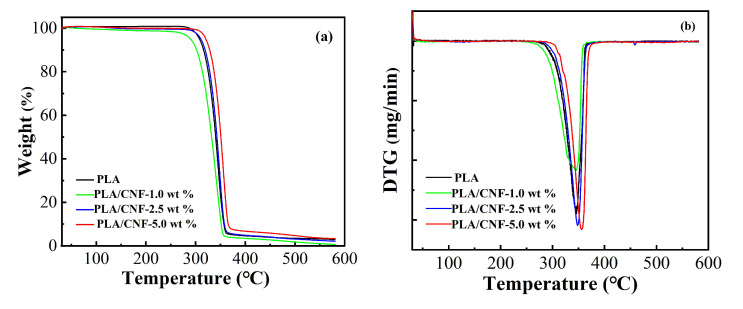
TGA (**a**) and DTG (**b**) graphs of PLA/CNF biocomposite films.

Both improvement and deterioration in the thermal stability of PLA by adding the nanocellulose from different sources were reported [21,31,32]. One of the purposes when incorporating CNF into PLA was to evaluate the effect of CNF on the thermal stability of PLA-based biocomposites. TGA and DTG (first derivative of the TGA) curves for pure PLA and PLA/CNF biocomposite films are shown in Figure 7a,b, respectively. Table 1 summarizes the thermal parameters, including T_onset_ (the temperature at which the weight loss starts), T_10%_ (the 10% weight loss temperature), T_50%_ (the 50% weight loss temperature), and T_max_ (maximum degradation temperature), respectively. Only a single degradation stage between 300 and 370 °C was observed for the neat PLA and PLA/CNF biocomposites. Surprisingly, the decomposition temperatures for PLA/CNF-1.0 wt % were lower than that of neat PLA, while the decomposition temperatures for PLA/CNF-2.5 wt % were just a little higher than that of pure PLA. Enhanced thermal stability was only detected for PLA/CNF-5.0 wt %. As compared to pure PLA, T_onset_ and T_max_ were increased by 20 °C and 10 °C, respectively. The enhancement in the thermal stability for PLA/CNF biocomposites could only be achieved when the content of the CNF was at 5.0 wt % in our case.

### 2.5. DSC Analysis of CNF Filled PLA Biocomposite Films

**Figure 8:**
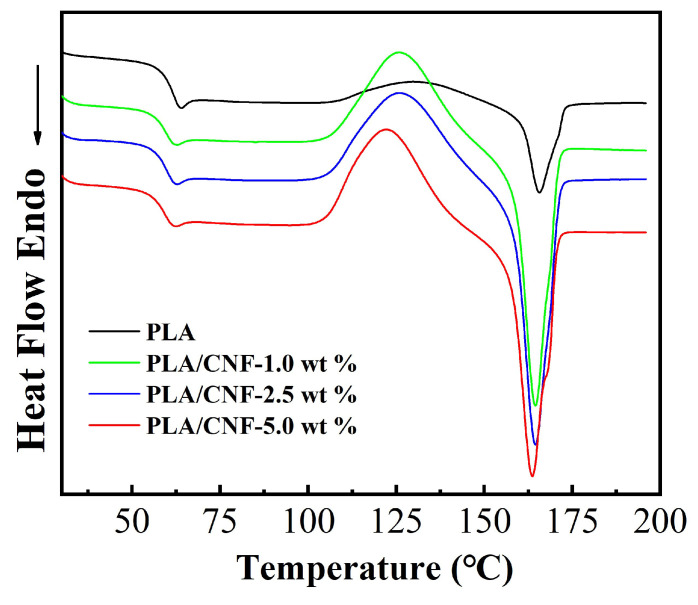
Differential scanning calorimetry (DSC) curves of second heating for neat PLA and PLA/CNF biocomposite films.

DSC analysis was conducted to find out possible changes in PLA thermal properties induced by the incorporation of CNF. The DSC curves of second heating for both neat PLA and PLA/CNF biocomposites are described in Figure 8 and the thermal parameters are listed in Table 2. In the DSC curves, all particular transitions of PLA, including T_g_ (glass transition temperature), T_cc_ (cold crystallization temperature), T_m_ (melting temperature), ΔH_cc_ (cold crystallization enthalpy), and ΔH_m_ (melting enthalpy) are listed, showing the difference of PLA/CNF biocomposites. The DSC curve of PLA/CNF composites showed similar trends but different thermal parameters as that of neat PLA. T_g_, T_cc_, and T_m_ of PLA/CNF biocomposites were lower than that of neat PLA film (Table 2). The T_g_ of PLA was 60.6 °C, while that of the PLA/CNF was slightly lower, which was consistent with the previous study [33]. PLA/CNF biocomposites started to crystallize easily than neat PLA films. The mobility of PLA chains was altered by the incorporation of CNF [34]. Slightly lower T_cc_ was detected for PLA/CNF biocomposite films. The decline in T_cc_ indicated that CNF worked as the nucleating agent and enhanced the crystallization process of the PLA/CNF biocomposites. The differences in T_m_ for neat PLA and PLA/CNF biocomposites were quite small (less than 1 °C) at around 165 °C. Both ΔH_cc_ and ΔH_m_ were shown to be increased compared with that of pure PLA, implying that overall the introduction of CNF enhanced the crystallinity of PLA biocomposite films.

### 2.6. Tensile Analysis of CNF Filled PLA Biocomposite Film

**Figure 9:**
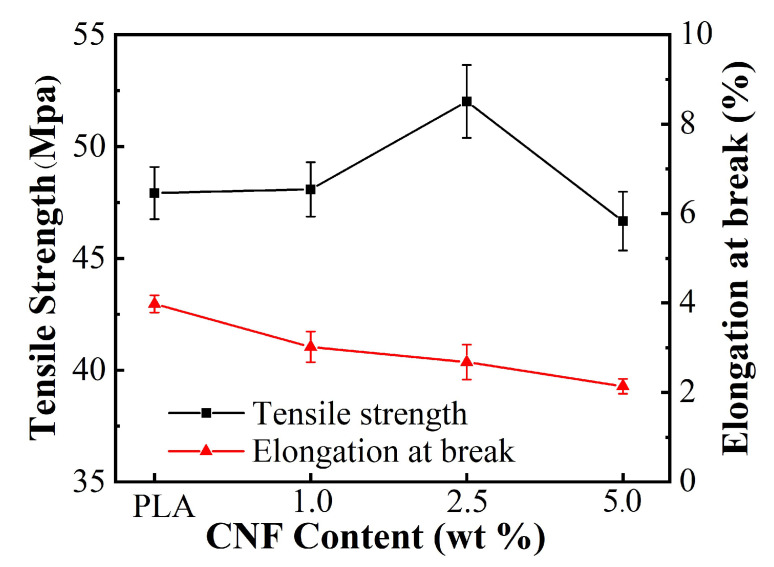
Tensile properties PLA/CNF biocomposite films with different CNF loading.

The tensile strength of neat PLA and PLA/CNF biocomposite films are displayed in Figure 9. As can be seen, the tensile strength of the PLA/CNF biocomposite increased first and then decreased, and the elongation at break gradually decreased with the increase in CNF contents. The tensile strength reached the maximum when the CNF loading was 2.5 wt %. The maximum strength was 52 MPa, which was 8.5% higher than that of pure PLA film. This enhancement in tensile strength was due to the good dispersibility and interfacial interactions of CNF in the PLA matrix. When the loading of CNF was lower (1.0 wt %) than the critical percolation threshold, the CNF network cannot form in the PLA matrix, so it had little effect on mechanical enhancement [35]. CNF may suffer from self-aggregation at 5.0 wt % in the PLA matrix, which results in reduced tensile strength for the biocomposite film. However, CNF reinforced PLA biocomposites exhibited elongation at break than that of pure PLA matrix. The decreases in elongation at break were also reported by Sung et al. [32,33]. The large standard deviations on the tensile strength were ascribed to the inhomogeneous distribution of CNF in the PLA matrix. This imperfect dispersion and interfacial adhesion can be used to explain the tensile strength differences between experimental data and theoretical predictions [26].

**Figure 10:**
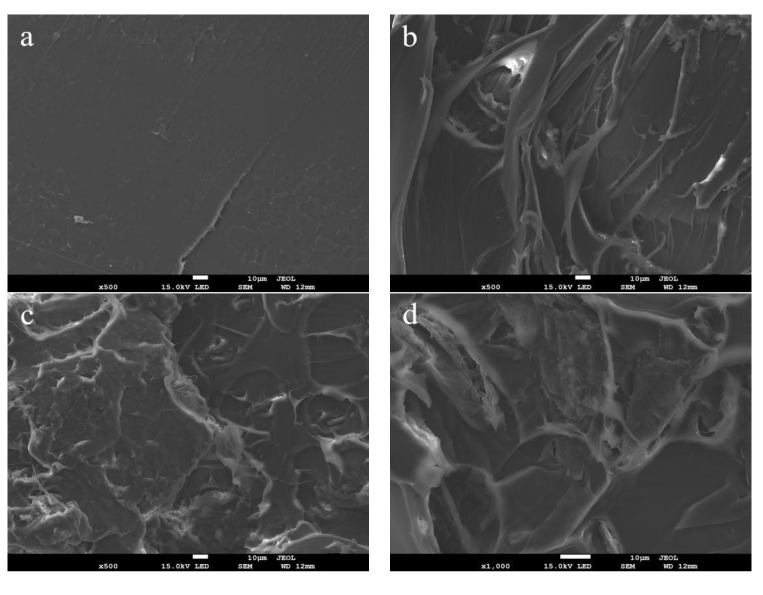
SEM image of cross-section at fracture for neat PLA film and PLA/CNF biocomposite films: (**a**) PLA; (**b**) PLA/CNF-1.0 wt %; (**c**) PLA/CNF-2.5 wt %; and (**d**) PLA/CNF-5.0 wt %.

The cross-section morphologies of PLA/CNF biocomposite materials at fracture after tensile testing were also analyzed as shown in Figure 10. The cross-section of neat PLA film was very clean and smooth, showing typical brittle fracture characteristics [36]. The cross-section of the PLA/CNF-1.0 wt %, PLA/CNF-2.5 wt %, and PLA/CNF-5.0 wt % showed irregular fibrous appearance after CNF incorporation, indicating that the dispersion of CNF in the PLA matrix largely changed the physical structures of the biocomposites. The surface of PLA/CNF-2.5 wt % exhibited a rougher surface as compared with that of PLA/CNF-1.0 wt % and PLA/CNF-5.0 wt %, which may explain the highest tensile strength measured for the PLA/CNF-2.5 wt % biocomposites. The result indicated that the more irregular the cross-section at fracture after tensile testing, the better tensile strength for the PLA/CNF biocomposites.

## 3. Materials and Methods

### 3.1. Materials

Microcrystalline cellulose (MCC) was obtained from Qufu Tianli Pharmaceutical Dressing Co, China. Polylactide (PLA, 4032D) obtained from Nature Works was used for the experiment. Commercial cellulase, Cellic^®^ CTec2, was obtained from Novozymes North America (Franklinton, NC, USA). Other chemical agents, such as acetic acid, sodium acetate, dichloromethane, and ethanol were all analytically pure from Sinopharm Group Chemical Reagent (Shanghai, China) and used as received.

### 3.2. CNF Isolation by Enzymatic Pretreatment and High-Pressure Homoginaztion

CNF was isolated following the protocol developed in our previous studies [34,37,38]. Briefly, enzymatic pretreatment of MCC was conducted at 10 wt % MCC loading, 12.8 FPU/g MCC, and 50 °C for 2 h in the acetic buffer (pH 4.6). The pretreated MCC was inactivated and thoroughly washed. Of the suspension 2 wt % was directly fed into high-pressure homogenizer (AH-Basic with an R-shaped chamber, Suzhou ATS Engineering, Suzhou, China). The fibrils were then passed through the homogenizer at 90 PMa up to 10 times. The obtained CNF were freeze-dried (Beta 1-8LD plus, Martin Christ, Osterode am Harz, Germany) and milled to pass through a 300-mesh.

### 3.3. Fabrication of PLA/CNF Biocomposite Films by Solution Cating and Melt Compression

PLA solution was prepared by dissolving PLA in dichloromethane. CNFs at 1.0 wt %, 2.5 wt %, and 5.0 wt % loadings respective to PLA were added to PLA solutions and thoroughly mixed. The mixture solutions were then cast in Petri dishes and PLA/CNF biocomposite films were obtained after the evaporation of dichloromethane. The obtained biocomposite films sandwiched between 2 layers of the thin steel plates and polyimide films were further hot-pressured at 175 °C and 50 kg/cm^2^ for 2 min. The obtained biocomposite films were labeled as PLA/CNF-1.0 wt %, PLA/CNF-2.5 wt %, and PLA/CNF-5.0 wt %, respectively.

### 3.4. CNF and PLA/CNF Biocomposite Film Characterization

#### 3.4.1. Scanning Electron Microscopy (SEM)

SEM observations of the freeze-dried CNF and cross-section of PLA/CNF biocomposite films were conducted with a JSM-7800F (SEM, JSM-7800F, Tokyo, Japan). The samples were sputter-coated with a thin layer of gold and the observation voltage was 15 kV.

#### 3.4.2. UV-Vis Transmittance

The UV-vis transmittance of the PLA biocomposite films with thickness of 0.1 mm was examined from 200 to 800 nm by a DU-800 UV-Vis spectrophotometer (Beckman Coulter, Brea, CA, USA).

#### 3.4.3. Fourier Transform Infrared Apectroscopy (FTIR)

FTIR experiments of neat PLA and PLA/CNF biocomposite films were performed with a Nexus 470 FTIR spectrometer (Nicolet Instruments, Dreieich, Germany). The spectra of PLA/CNF biocomposite films were recorded from 4000 to 500 cm^−1^.

#### 3.4.4. X-ray Diffraction (XRD)

XRD profiles of freeze-dried CNF powder and PLA/CNF films were obtained by using a D8 Advance X-ray powder diffractometer operated at 30 kV, 15 mA, and 2°/min in the range of 2*θ* = 4° to 2*θ* = 40°.

#### 3.4.5. Tensile test

Tensile tests were examined using a tensile tester (JF-9003; Dongguan Jianfeng Instrument Limited, Guangdong, China) with a span of 50 mm, and an elongation speed of 5 mm/min was adopted. Each sample was determined five times and the average data were reported.

#### 3.4.6. Thermogravimetric Analysis (TGA)

Thermal property analysis of neat PLA and PLA/CNF film (4–6 mg) was examined with a TGA4000 thermogravimetric analyzer (Perkin-Elmer, Waltham, MA, USA) with a heating rate of 10 °C/min from 30 to 600 °C under nitrogen environment (20 mL/min).

#### 3.4.7. Differential Scanning Calorimetry (DSC)

DSC experiments were examined with a DSC4000 instrument in a nitrogen atmosphere from 30 to 200 °C. The heating and cooling rates to eliminate the influence of thermal history were 10 °C/min and 100 °C/min from 30 to 200 °C and from 200 to 30 °C, respectively. The DSC profiles of PLA/CNF biocomposite films were obtained at a heating rate of 10 °C/min from 30 to 200 °C after removing the thermal history.

## 4. Conclusions

In this study, CNF reinforced PLA biocomposites were fabricated by solution casting and melt compression. The structure and properties of PLA/CNF biocomposite films were investigated with the aids of UV-vis spectrophotometer, ATR-FTIR, XRD, TGA, DSC, and tensile tester. The tensile strength of PLA/CNF biocomposites was improved with 2.5 wt % CNF and then decreased when the CNF content was 5.0 wt %. According to the results of TGA, the thermal stability of PLA/CNF biocomposites improved substantially at the 5.0 wt % CNF loading. The PLA/CNF-5.0 wt % biocomposite showed a 20 °C increase in T_onset_ and a 10 °C increase in T_max_ over the neat PLA matrix, respectively. Additionally, the cold crystallization temperature (T_cc_) increased by 5.6 °C for PLA/CNF-5.0 wt % over the neat PLA film. Overall, CNF reinforced PLA biocomposites showed good physical properties. However, the practical transition of the fabrication of PLA/CNF biocomposites from laboratory to industrial scale is still challenging. Further studies are required to improve the technology and engineering processes.

## Figures and Tables

**Table 1:** TGA thermal characteristics of PLA and PLA/CNF biocomposite films.

Sample	T_onset_ (°C)	T_10%_ (°C)	T_50%_ (°C)	T_max_ (°C)
PLA	311	316	340	346
PLA/CNF-1.0 wt %	304	302	332	343
PLA/CNF-2.5 wt %	320	319	343	348
PLA/CNF-5.0 wt %	331	328	351	356

**Table 2:** DSC thermal characteristics of PLA and PLA/CNF nanocomposites.

Sample	T_g_ (°C)	T_cc_ (°C)	T_m_ (°C)	ΔH_cc_ (J/g)	ΔH_m_ (J/g)
PLA	60.6	128.9	165.0	9.1	10.8
PLA/CNF-1.0 wt %	59.6	125.8	164.4	36.9	37.4
PLA/CNF-2.5 wt %	59.2	125.6	164.6	40.3	41.2
PLA/CNF-5.0 wt %	59.4	122.3	164.7	38.1	38.9
